# Polymorphisms in the *CYP2E1* and *GSTM1* Genes as Possible Protection Factors for Leprosy Patients

**DOI:** 10.1371/journal.pone.0047498

**Published:** 2012-10-15

**Authors:** Pablo Pinto, Claudio Guedes Salgado, Ney Santos, Dayse O. Alencar, Sidney Santos, Mara H. Hutz, Ândrea Ribeiro-dos-Santos

**Affiliations:** 1 Laboratório de Genética Humana e Médica, Instituto de Ciências Biológicas, Universidade Federal do Pará, Belém, Pará, Brasil; 2 Laboratório de Dermatoimunologia, Instituto de Ciências Biológicas, Universidade Federal do Pará, Belém, Pará, Brasil; 3 Instituto de Biociências, Departamento de Genética, Universidade Federal do Rio Grande do Sul, Rio Grande do Sol, Brasil; Queen Mary University of London, United Kingdom

## Abstract

**Background:**

The *CYP2E1* and *GSTM1* genes encode metabolic enzymes that have key functions in drug modification and elimination.

**Methodology/Principal Findings:**

We investigated the possible effects of *CYP2E1* and *GSTM1* polymorphisms in 71 leprosy patients and in 110 individuals from the general population. The GSTM1*0 null allele and INDEL CYP2E1*1D mutant genotypes were analyzed by conventional PCR, while *CYP2E1* SNPs (1053C>T, 1293G>C and 7632T>A) were determined by RT-PCR. In leprosy patients, the GSTM1*0 and CYP2E1*5 alleles and the combined alleles GSTM1*0/CYP2E1*6 and GSTM1*0/CYP2E1*5 were significantly related to a baciloscopic index (BI) (BI<3), while the CYP2E1*6 allele was related to a better clinical evolution in the leprosy spectrum.

**Conclusions/Significance:**

Therefore, GSTM1*0, CYP2E1*5 and CYP2E1*6 may be possible protection factors for leprosy patients.

## Introduction

Leprosy is an insidious infectious disease caused by the obligate intracellular bacteria *Mycobacterium leprae* that affects the skin and peripheral nerves, causing a chronic granulomatous infection [1].

Multidrug therapy (MDT), the treatment recommended by the World Health Organization (WHO), has healed millions of patients since it was implemented in 1980s. MDT consists of the use of dapsone and rifampicin for 6 months in paucibacilary (PB) patients, or both along with a third drug, clofazimine, for 12 months in multibacilary (MB) cases [2].

Patients are classified as PB or MB using a simple system introduced by the WHO in 1982. Patients with 5 skin lesions or less are classified as PB, and those with more than 5 lesions are classified as MB [3], [4]. Although this simple classification scheme is adequate for remote sites where the population has little or no access to health care, it is not detailed enough for more in-depth research surveys.

Another way to classify leprosy patients is based on a skin smear test, for which a positive result is classified as MB, and a negative result as PB. A trained laboratory technician can readily identify AFB (acid fast bacillus), making this test a very reliable method. However, cases initially classified as PB (AFB negative) can evolve to MB in the natural course of the disease [5]. This phenomenon is especially true for those patients classified as indeterminate (MHI) using the Madrid classification system.

The use of the Ridley-Jopling clinical, histological and immunological criteria further improves case definitions, with TT (tuberculoid-tuberculoid) patients exhibiting a strong cellular immune response (CIR) and a negative skin smear test, while LL (lepromatous-lepromatous) patients have a weak or absent CIR and a highly positive skin smear [6]. In the middle of the spectrum are a large number of borderline patients, varying from weak to strong CIR and from negative to positive skin smears.

Interestingly, neither the CIR status nor the skin smear test is predictive of leprosy reactions or of the progression of each case through the physical disabilities caused by the disease. To date, little is known about which factors are crucial to the development of these disabilities. All types of patients, TT, borderline or LL, can progress with highly incapacitating disabilities and chronic neuropathies with no single marker or criteria to predict patient outcome [2], [3].

Dapsone (4,4′-diaminodiphenyl sulfone, DDS) is one of the primary drugs used in anti-leprosy therapeutics. It is a bacteriostatic agent that competes with para-aminobenzoic acid (PABA), diminishing or blocking the production of bacterial folic acid [5], [7]. Clofazimine is a riminophenazine dye that has bactericidal and anti-inflammatory effects. It inhibits bacterial proliferation by binding to bacterial DNA and blocking its replication [8], [9]. Rifampicin, or rifampin (RIF) has a well-proven bactericidal effect on *M. leprae*. It is a semisynthetic drug, originally derived from *Streptomyces mediterranei*, and it is widely used for treating leprosy and tuberculosis. RIF prevents protein production by inhibition of RNA polymerase in bacterial cells [10]–[16].

CYP-450 members of the heme protein superfamily are notable for their large spectrum of action and the distribution of their biological structures. These proteins participate in critical processes including the biosynthesis of steroidal hormones and the detoxification by conjugation with cellular components, such as glutathione [17].

CYP2E1 is found in various tissues including brain, lungs and kidneys, but it is most concentrated in the liver, where the majority of biotransformation occurs. Four main SNPs in the *CYP2E1* gene have been investigated in different populations, including tuberculosis (TB) patients [18]–[20], who often show 1053C>T and 1293G>C mutations, which together form the compound allele CYP2E1*1A, CG; CYP2E1*5, TC. Another SNP, 7632T>A, is located in the sixth intron of the *CYP2E1* gene and has two alleles, wild-type CYP2E1*1A (T) and mutant CYP2E1*6 (A). Additionally, a 96-bp INDEL polymorphism with two alleles, wild-type CYP2E1*1C (DEL) and mutant CYP2E1*1D (INS), has also been described [18], [19].

There are different glutathione S-transferase (GST) isoforms, including *GSTM1*, which is located on chromosome 1. More than 51 SNPs have been described within *GSTM1*, among which are two functional alleles, GSTM1*A and GSTM1*B, that have the same detoxification efficacy; one null (deletion) allele, GSTM1*0; and one duplication [21], [22].

Two GSTM1 polymorphisms, GSTM1*1, which has normal activity, and GSTM1*0, which has no enzymatic activity because it is a complete gene deletion, have been well studied in different populations [23]–[25]. The presence of the null allele seems to be related to substrate conjugation and excretion; therefore, its presence can be an indicator for more rational drug dosages for various groups of patients [26].

We investigated a sample of MDT-treated leprosy patients ascertained at the Dr. Marcello Candia Reference Unit in the Sanitary Dermatology of the State of Pará (UREMC) with the aim of identifying associations among ***CYP2E1*** polymorphisms [including 1053 C>T, 1293G>C (CYP2E1*1A, CYP2E1*5); 7632T>A (CYP2E1*1A, CYP2E1*6); 96-bp INDEL CYP2E1*1C (DEL) and CYP2E1*1D (INS)] and ***GSTM1*** polymorphisms (GSTM1*1 and GSTM1*0) and possible protection factors for leprosy patients.

## Methods

### Sample

We investigated 71 leprosy patients who attended the Dr. Marcello Candia Reference Unit in the Sanitary Dermatology of the State of Pará (UREMC) in Marituba, Pará, Brazil, from January 2008 to December 2009. In UREMC there are about 40,000 yearly consultations on different medicine specialties as, among others, leprology, dermatology, ophthalmology and orthopedics, besides nursery, physical therapy and other health professionals sessions. Since 2002, UREMC registered between 308 and 472 leprosy patients (mean: 408 cases/year). During years 2008 and 2009, 765 leprosy cases were registered, from those, 71 (9,28%) were randomly selected for this study.

All patients were evaluated neurologically by Semmes-Weinstein monofilament examination (SWME) for sensory testing, and by voluntary motor testing (VMT) for function assessment of muscular force, as previously described [27]. They were classified according to the Ridley-Jopling system and were distributed in two groups depending on the progression of the disease: the positive (+) group consisted of PB patients skin smear negative Tuberculoid (TT) patients and MB skin smear negative Borderline-Tuberculoid (BT) patients, with or without leprosy reactions, with no sequel (defined by sensorial loss or motor deficit on hands or feet on Nerve Function Impairment (NFI) assessment), and the negative (−) group consisted of PB patients with or without leprosy reactions, but with sequel, together with all MB skin smear positive Borderline-Borderline (BB), Borderline-Lepromatous (BL) and Lepromatous-Lepromatous (LL) patients, regardless reactions or sequel. Additionally, in order to make other comparisons, patients were classified according to the baciloscopic index (BI), a group with a low (<3) BI (LBI), and a group with high (≥3) BI (HBI) [28]. A sample of 110 healthy individuals from the same geographic area were included in the study as controls. All patients were informed about the study before signing informed consent forms. The project was approved by the Pará Federal University ethics committee (N° 197/07).

### DNA Extraction

DNA extraction was performed as previously described [29]. The DNA concentration was determined by spectrophotometry (Themo Scientific NanoDrop 1000, NanoDrop Technologies, Wilmington, US).

### CYP2E1 Genotyping

Three *CYP2E1* polymorphisms, 1053C>T, 1293G>C and 7632T>A, were investigated using a TaqMan genotyping assay and analyzed by Real Time PCR 7500 (Life Technologies, CA, USA). The INDEL was investigated using conventional PCR methods, followed by visualization on an agarose gel. Specific PCR programs were established according to the annealing temperatures of the primers, and the amplifications were performed on a thermocycler Veriti 96 Well Thermal Cycler (Life Technologies, CA, USA). The alleles of the four *CYP2E1* polymorphisms investigated were defined using the official nomenclature, as described in http://www.cypalleles.ki.se/cyp2e1.htm.

### GSTM1 Genotyping

For amplification, a set of primers for GSTM1F/GSTM1R was investigated using conventional PCR methods (thermocyclerVeriti 96 Well Thermal Cycler - Life Technologies, CA, US), followed by visualization on an agarose gel.

### Ancestry Informative Markers (AIM)

Individual interethnic admixture was estimated using a panel of 48 ancestry informative markers (AIMs), as previously described [30].

### Statistical Analyses

Estimations of linkage disequilibrium (D and D’) and haplotypes and allelic frequencies were estimated with the M. Locus v. 2.0 software [31]. All other statistical analyses were performed using SPSS v. 12.0 (SPSS, Chicago, IL, USA), and results were considered statistically significant at p<0.05.

## Results

Demographic and clinical characteristics of the patients are shown in Table 1. Age, gender, sequel and clinical forms were all statistically significant when LBI and HBI were compared. Sequel occurred in 76.9% of the HBI patients whereas in the LBI group only 37.5% of the patients presented sequel.

**Table 1:** Demographic and clinical characteristic of the sample according with Baciloscopic Index.

Variables	Baciloscopic Index^c^ (N = 71)		*p value* (IC-95%)
	LBI n(%) = 32	HBI n(%) = 39	
Age^a^	35.2± 2.97	62.5± 3.42	<0.001
Gender^b^ (M/F)	15(46.8%)/17(53.2%)	32(82%)/7(12%)	0.003
Sequel^b^ (YES/NO)	12(37.5%)/20(62.5%)	30(76.9%)/9(23.1%)	0.001
Clinical Forms (PB/MB)^b^	10(31.2%)/22(68.8%)	0/39(100%)	0.002

a *t*-Test of Student;

b Fisher’s Exact Test;

c Baciloscopic Index (LBI  =  Baciloscopic Index Low; HBI  =  Baciloscopic Index High).

There were 19 patients classified in the positive (+) group, of which nine were designated PB (two with reaction) and 10 were MB (four with reaction and all without sequel), while 52 patients comprised the negative (−) group, two of which were PB (all with reaction and sequel), and 50 were MB (35 with reaction and 40 with sequel) (Table 2). Concerning genotypic and allelic distribution of SNPs, a high (42.3%), statistically significant, frequency of the heterozygous genotype for the CYP2E1*6 allele was found among leprosy patients (Table 2). In the positive group, 63.2% (12 patients) exhibited this genotype, while in the negative group, a lower percentage (34.6%, 18 patients) was observed. The frequency of the wild-type CYP2E1*1A and mutant CYP2E1*6 alleles in this population was 0.789 and 0.211, respectively, which was statistically significant when the positive and negative groups were compared (Table 2).

**Table 2:** Genotypic and allelic distribution of SNPs on *CYP2E1* and *GSTM1* genes among patients grouped according to clinical evolution.

Genotype	Patients with Leprosy (%) (n = 71)	Group (+) (%) (n = 19)	Group (−) (%) (n = 52)	P* ^1^	OR(95% IC)* ^2^
***CYP2E1 (96 INDEL)***					
* **1C/** * **1C**	64 (90.1%)	16 (84.2%)	48 (92.3%)		1 (reference)
* **1C/** * **1D**	7 (8.9%)	3 (15.8%)	4 (7.7%)	0.375	0.444(0.09–2.202)
***CYP2E1*** * ***1C***	0.951	0.921	0.962		
***CYP2E1*** * ***1D***	0.049	0.079	0.038		
***CYP2E1 (7632)***					
* **1A/** * **1A**	41 (57.7%)	7 (36.8%)	34 (65.4%)		1 (reference)
* **1A/** * **6**	30 (42.3%)	12 (63.2%)	18 (34.6%)	**0.03**	0.309(0.103–0.922)
***CYP2E1*** * ***1A***	0.789	0.684	0.827		
***CYP2E1*** * ***6***	0.211	0.316	0.173		
***CYP2E1 (1053/1293)***					
* **1A/** * **1A**	56 (78.9%)	13 (68.5%)	43 (82.6%)		1 (reference)
* **1A/** * **5**	15 (21.1%)	6 (31.5%)	9 (17.4%)	0.206	0.453(0.135–1.513)
***CYP2E1*** * ***1A***	0.894	0.842	0.914		
***CYP2E1*** * ***5***	0.106	0.158	0.086		
***GSTM1***					
***GSTM1*** * ***1***	38 (53.5%)	12 (63.2%)	26 (50%)		1 (reference)
***GSTM1*** * ***0***	33 (46.5%)	7 (36.8%)	26 (50%)	0.423	1.714 (0.583–5.043)

* ^1^p-value;

* ^2^OR-odds ratio, CI-confidence interval.

Leprosy patients were also divided into two groups according to the baciloscopic index (BI): a group with a low (<3) BI (LBI) and a group with high (≥3) BI (HBI). In addition to the analysis of the genotypic distribution of both *CYP2E1* and *GSTM1* markers in the LBI and HBI groups (Table 3), the combined effect of the two mutant alleles for the *CYP2E1* and *GSTM1* genes (CYP2E1*6/GSTM1*0 and CYP2E1*5/GSTM1*0) was also analyzed. The mutant CYP2E1*5 allele was present in 37.5% of the patients in the LBI group, while the wild-type CYP2E1*1A allele was observed in 92.3% of the patients in the HBI group. *GSTM1* gene analysis demonstrated that the mutant GSTM1*0 allele was present in 56.2% of the LBI group patients and in 38.5% of the HBI group patients, while the wild-type GSTM1*1 allele was present in 61.5% of the HBI group patients (Table 3). The analysis of the combined effect revealed that the CYP2E1*6/GSTM1*0 genotypic combination was detected in 31.2% of the patients in the LBI group, while the CYP2E1*5/GSTM1*0 genotypic combination was present in 28.1% of the patients in the LBI group; all were statistically significant when the different combinations were analyzed in the LBI or HBI groups.

**Table 3:** Combined and isolated genotypic distribution of *CYP2E1* gene (SNPs 1053T>C, 1293C>G and 7632T>A), and deletion (*GSTM1* * *1/GSTM1* * *0*) on gene *GSTM1* of patients classified accordingly to baciloscopic index BI (LBI and HBI).

Genotype	Leprosy patients (n = 71)	LBI (n = 32)	HBI (n = 39)	p* ^1^	OR (95% IC)* ^2^
***CYP2E1*** ** (7632)**					
* ***1A/*** * ***1A***	41 (57.74%)	16 (50%)	25 (64.1%)		1 (reference)
* ***1A/*** * ***6***	30 (42.26%)	16 (50%)	14 (35.9%)	0.334	0.560 (0.216–1.452)
***CYP2E1*** * ***1A***	0.789	0.750	0.821		
***CYP2E1*** * ***5***	0.211	0.250	0.179		
***CYP2E1 (*** **1053/1293)**					
* ***1A/*** * ***1A***	56 (78.87%)	20 (62.5%)	36 (92.3%)		1 (reference)
* ***1A/*** * ***5***	15 (21.13%)	12 (37.5%)	3 (7.7%)	**0.003**	0.139 (0.035–0.551)
***CYP2E1*** * ***1A***	0.894	0.813	0.962		
***CYP2E1*** * ***5***	0.106	0.187	0.038		
***GSTM1***					
***GSTM1*** * ***1***	38 (53.52%)	14 (43.8%)	24 (61.5%)		1 (reference)
***GSTM1*** * ***0***	33 (46.48%)	18 (56.2%)	15 (38.5%)	**0.0276**	0.486 (0.188–1.258)
***CYP2E1/GSTM1*** * **^3^**					
***CYP2E1*** * ***1A/GSTM1*** * ***1***	58 (81.7%)	22 (68.8%)	36 (92.3%)		1 (reference)
***CYP2E1*** * ***6/GSTM1*** * ***0***	13 (18.3%)	10 (31.2%)	3 (7.7%)	**0.012**	0.183(0.045–0.740)
***CYP2E1*** * ***1A/GSTM1*** * ***1***	62 (87.3%)	23 (71.9%)	39 (100%)		1 (reference)
***CYP2E1*** * ***5/GSTM1*** * ***0***	9 (12.7%)	9 (28.1%)	–	**<0.005**	0.371 (0.27–0.513)

* ^1^p-value;

* ^2^OR-odds ratio, CI-confidence interval;

* ^3^Combined effect of mutant alleles of distinct genes.

Next, we performed a logistic regression analysis in which the two groups, LBI and HBI, were dependent variables and with covariables that could interfere with the results of PB and MB clinical forms. Although the results were not statistically significant for different variables, such as gender and sequel, they were significant when related to CYP2E1*1A/*5 (p = 0.0266) and GSTM1*0 (p = 0.0500) genotypes. These results suggest a strong association between both mutations and LBI (Table 4).

**Table 4:** Logistic regression analysis of the association between genetic markers and LBI/HBI response in leprosy patients.

Variable	β	S.E.	Wald	df	*P*	O.R (95%CI)
Age	0.0363	0.0209	3.0109	1	**0.0012**	1.0562 (0.9953–1.0804)
Gender	0.8705	0.7327	1.4113	1	0.2348	2.3881 (0.5680–1.0407)
Sequel	0.8742	0.8405	1.0818	1	0.2983	2.3969 (0.4616–12.4468)
Clinical Form(PB/MB)	1.8598	1.1901	2.4421	1	0.1181	6.4226 (0.6233–66.1807)
*CYP2E1* *1A/*5	−1.6341	0.8457	3.7339	1	**0.0266**	0.1198 (0.0184–0.7816)
*CYP2E1* *1A/*6	1.0889	0.9079	1.4382	1	0.2304	2.9709 (0.5012–17.6087)
GSTM1*0	−1.3004	0.7025	3.4262	1	**0.05**	0.2724 (0.0687–1.0796)
African	−2.2083	1.8217	3.3266	1	0.4925	0.1099 (0.0002–60.2213)
European	0.9890	2.6275	0.1417	1	0.7066	2.6885 (0.0156–10.6548)
Amerindian	0.4674	2.5465	6.5769	1	0.8544	1.5958 (0.0108–11.0136)

**β**, Coefficient Stimation; **S.E.**, Standard Error; **df**, Degrees of Freedom; ***p***, p-value; **OR**, Odds Ratio; **CI**, Confident Interval.

**Table 5:** Allele and genotype distributions of CYP2E1 and GSTM1 genes within two samples from leprosy patients and healthy individuals.

Genotype	Patients with Leprosy (%) (n = 71)	Healthy Population (%) (n = 110)	?^2^	p
***CYP2E1 (96 INDEL)***				
*1C/*1C	64 (90.1%)	96 (87.3%)		
*1C/*1D	7 (8.9%)	12 (10.9%)		
*1D/*1D	–	2 (1.8%)		
CYP2E1*1C	0.951	0.927	1.376	0.743
CYP2E1*1D	0.049	0.073		
***CYP2E1 (7632)***				
*1A/*1A	41 (57.7%)	90 (81.8%)		
*1A/*6	30 (42.3%)	20 (17.2%)		
*6/*6	–	–		
CYP2E1*1A	0.789	0.909	11.673	**0.003**
CYP2E1*6	0.211	0.091		
***CYP2E1 (1053/1293)***				
*1A/*1A	56 (78.9%)	70 (63.6%)		
*1A/*5	15 (21.1%)	37 (33.6%)		
*5/*5	–	3 (2.8%)		
CYP2E1*1A	0.894	0.805	6.855	**0.032**
CYP2E1*5	0.106	0.195		
***GSTM1***				
GSTM1*1	38 (53.5%)	53 (48.2%)		
GSTM1*0	33 (46.5%)	57 (51.8%)	1.136	0.722

To evaluate the presence of population substructure, we compared the clinical progression of leprosy patients (positive and negative groups, as well as high and low baciloscopic index groups) with genomic ancestry, and the results showed no, significant. However, different frequencies were found for the investigated markers when leprosy patients were compared with a sample of healthy individuals from the same region (Table 5). The data showed that CYP2E1*5 allele is more frequent among the healthy individuals than among patients (0.196 and 0.106, respectively; X^2^ = 6.85; p = 0.032), while CYP2E1*6 allele is more common among patients than in the control sample (0.211 e 0.090, respectively; X^2^ = 11.6; p = 0.003).

## Discussion

Loss of sensation is the hallmark of leprosy diagnosis. It is well known that both, MB and PB patients may evolve to nerve function impairment on the natural course of the disease [32]. It is usual - and comprehensible as an objective tool – to use BI to analyze the correlation between a specific gene or a genotypic combination and the evolution of leprosy. However, this cannot be the only parameter to evaluate in order to understand the disease behavior individually. HBI may indicate *M. leprae* ability to grow inside the host in order to keep transmission chain and strain survival, or may indicate the inability of the host in constrain bacterial growth.

Notwithstanding, the capacity of the human host immune system in dealing with leprosy infection with no sequel is rarely addressed. In the present study two groups of patients were examined, and a striking difference when BI or disease evolution were evaluated were observed in relation to the genes investigated herein. While CYP2E1^7632^*1A/*1A was associated to a worse disease progression, and the presence of the mutant CYP2E1^7632^*1A/*6 was associated with a good evolution, however, none of them were related to LBI or HBI. These findings suggest that different genes may be related to disease progression or bacterial growth inhibition mechanisms. Furthermore, CYP2E1^1053/1293^*1A/*1A was associated with HBI, while there no significant association was observed for clinical evolution analyses., CYP2E1^1053/1293^*1A/*5 was significantly associated with LBI and a better disease progression.

The availability of modern antibiotics can help us to better understand the disease, and it is reasonable to think that pharmacogenomics related genes may also be related to disease outcome in human hosts. One of these key drugs is rifampicin.

Rifampicin can be bacteriostatic at lower concentrations or bactericidal at higher concentrations. When used alone, mycobacterium can readily develop resistance to RIF, and therefore, treatment should not rely solely on this drug [15]. Its biotransformation occurs through a process of hepatic deacetylation, giving rise to the active metabolite desacetylrifampicin [26]. RIF has a high capacity for inducing CYP450 isoforms, which contributes to a 40% reduction in half-life during the first half month of treatment and the acceleration of RIF deacetylation. Therefore, this drug is capable of intensifying its own biotransformation, diminishing its plasmatic half-life when administered in multiple doses [33]. Studies of the *CYP2E1* gene indicate that CYP2E1*6 and CYP2E1*5 alleles are associated with a higher level of transcription and microsomal enzyme activity; therefore, they are implicated in enzymatic biotransformation activity augmentation [34]–[36], consequently decreasing the half-life of RIF.

Our results show that among the patients grouped according to clinical progression, the heterozygous genotype CYP2E1*1A/*6 was present in 63.2% of the individuals in the (+) group. The OR analysis of the CYP2E1*6 allele demonstrated that this polymorphism provides protection to those individuals in the (+) group (Table 2).

We hypothesize that the CYP2E1*6 allele could increased the rate of rifampicin metabolism. Augments the biotransformation by CYP450 enzymes and raising the levels of the active metabolite desacetylrifampicin, which has a higher bactericidal activity. Therefore, individuals with this mutation could more efficiently combat *M. leprae*.

A significant difference was found between healthy individuals and patients for the CYP2E1*6 allele, which is more common among leprosy patients (X^2^ = 11.6; p = 0.003). Since this association was unknown, more studies are necessary to confirm these results (Table 5).

Among leprosy patients, CYP2E1*5 allele was more frequent in the LBI group. This allele was also more frequent in healthy subjects when compared to leprosy individuals. These results taken together suggest that CYP2E1*5 is a protection factor that might be involved with bacterial growth inhibition (Table 5).

For the *GSTM1* gene, the null genotype GSTM1*0 was present in 56.2% of the LBI group. The compound distribution of the two mutant alleles CYP2E1*5/GSTM1*0 was present in 28.1% of LBI the patients, while CYP2E1*6/GSTM1*0 was present in 31.2%. The estimated OR suggest that mutant alleles confer protection for LBI individuals.

Taken together, our results suggest that the CYP2E1*5, CYP2E1*6 and GSTM1*0 alleles may be considered as susceptibility markers for leprosy, and their distribution should be further investigated, as their presence seems to confer protection from *M. leprae*.
